# Differential effects of hypnotherapy and cognitive behavioral therapy on the default mode network of depressed patients

**DOI:** 10.3389/fpsyg.2024.1401946

**Published:** 2024-06-27

**Authors:** Alina Haipt, David Rosenbaum, Kristina Fuhr, Anil Batra, Ann-Christine Ehlis

**Affiliations:** ^1^Department of Psychophysiology and Optical Imaging, University Hospital of Tuebingen, Tuebingen, Germany; ^2^Department of Psychiatry and Psychotherapy, University Hospital of Tuebingen, Tuebingen, Germany

**Keywords:** hypnotherapy, hypnosis, cognitive behavioral therapy, default mode network, depression, functional near-infrared spectroscopy

## Abstract

Hypnosis has been applied in healing procedures since the earliest of recorded history and today it is implemented in a wholesome concept Hypnotherapy (HT^1^). On a neurophysiological level, hypnosis has been associated with parts of the Default Mode Network (DMN^2^), but its effects on this network when induced in a treatment setting of a widespread disorder, namely depression, have never been investigated. Depression is associated with abnormal functional connectivity (FC^3^) of the DMN. Cognitive Behavioral Therapy (CBT^4^) has proven itself to be an effective treatment for depression; effects of CBT on DMN-related regions are heterogeneous. In the past years, HT was found to be a promising alternative or helpful adjunction. Yet, its underlying mechanisms remain to be unclear. In this original study 75 depressed patients receiving either CBT or HT were included and measured during resting-state before and after therapy with functional near-infrared-spectroscopy (fNIRS^5^). On symptom level, results show a significant reduction in both groups. On a neurophysiological level, first exploratory analyses hint toward treatment effects in two components of the DMN. However, these effects do not withstand correction for multiple testing. Still, our study is a first stepstone in the investigation of neural mechanisms of HT and offers first ideas about possible implications.

## 1 Introduction

Hypnosis and hypnotic trance have been used in healing rituals and practices since the earliest of recorded history (Hammond, 2013). In nowadays treatments it is applied in the medical and psychological field, often as adjunction to established treatment procedures. In this so-called Hypnotherapy (HT), states of trance, that are induced by hypnosis, are used to create an altered state of consciousness (Revenstorf, 2006), which is characterized by focused attention and reduced peripheral awareness (Griffiths, 2017). At the same time, hypnotic trance has been identified as a multi-facetted phenomenon, being linked to biological, psychological, and social factors (Jensen et al., 2015) which when it is applied in therapy, patients can learn to control symptoms and physiological functions that are usually not accessible consciously (Whorwell, 2011) and access individual resources (Revenstorf and Peter, 2009).

On a neurophysiological level, hypnosis and trance have been associated with changes in the Default Mode Network (DMN; Halsband and Wolf, 2021). The DMN is a core-network that was found to show robust coupling of spontaneous fluctuations (Greicius et al., 2008; Horovitz et al., 2009; Vanhaudenhuyse et al., 2010; Menon, 2011). The DMN is typically deactivated during attention demanding tasks but active during resting state (Raichle et al., 2001; Greicius et al., 2003). Its core nodes include the posterior cingulate cortex (PCC^6^), including the precuneus (PCu^7^), medial prefrontal cortex, nodes in the medial temporal lobe and the angular gyrus (Menon, 2011). The blood oxygen level dependent (BOLD^8^) signal shows a pattern of very low frequency range (>0.1 Hz; Sonuga-Barke and Castellanos, 2007). The DMN is associated with self-referential mental processes like thinking about one’s future, theory of mind, and affective decision making (Andrews-Hanna et al., 2010; Menon, 2011) and spontaneous thoughts during these self-referential processes (Mason et al., 2007; Rosenbaum et al., 2016, 2017).

The results to whether DMN activity during hypnosis increases or decreases vary among the studies. In earlier studies, increases in DMN activity during hypnosis were mainly found in the PCC and PCu as well as prefrontal areas like the anterior cingulate cortex (ACC^9^) (Rainville et al., 2002; Egner et al., 2005; Cojan et al., 2009; Pyka et al., 2011). Whereas later data suggests decreased activity in DMN associated regions, namely the ACC, PCC, and other prefrontal areas (McGeown et al., 2009; Deeley et al., 2012). Heterogeneity in these studies could result from the dissimilarities in study designs, tested samples, contents of hypnosis and types of suggestion. In a review of the existing literature the authors conclude on a relative consensus on decreased DMN functional connectivity (FC) during hypnosis/hypnotic trance while changes in the FC between the DMN and other core networks diverge (Halsband and Wolf, 2021).

Yet, the underlying neural mechanisms of hypnosis/hypnotic trance in a therapeutic context, thus HT, have only been investigated twice, namely in dental phobia (Lowén et al., 2013; Halsband and Wolf, 2015) and irritable bowel syndrome (Lowén et al., 2013), two disorders relatively uncommon [prevalence rate of 1.34% in German population (Häuser et al., 2019) and 3.7% of the Dutch population (Oosterink et al., 2009), respectively] compared to highly-prevalent disorders—like depression.

Depression is a widespread disease; currently approximately 280 million suffer from it according to the WHO (2021). Key symptoms in depression are a persistent sad mood, feelings of worthlessness and a loss of joy and interests (American Psychological Association, 2013). Additionally, depressed people were found to be impaired in affective cognition, e.g., the memory of emotional content (Elliott et al., 2011) and depressive rumination. The latter is defined as thoughts that focus on depressive symptoms and their implications (Nolen-Hoeksema, 1991). On a neurophysiological level, depression has been associated with alterations in the DMN, possibly mirroring depressive rumination on a symptom level (Berman et al., 2011; Rosenbaum et al., 2017). However, results on altered DMN activity in depression are slightly heterogeneous.

Hyperactivity in the DMN was found in depressed patients in several early studies (Anand et al., 2005; Greicius et al., 2007; Grimm et al., 2009; Sheline et al., 2009) and was discussed to account for impairments associated with depressive symptoms like emotional processing and cognitive performance (Drevets et al., 2008; Kaiser et al., 2015) or automatic affective processing (Sheline et al., 2009). Yet, in contrast, a depression-specific DMN decrease of FC was also observed (Connolly et al., 2013; Rosenbaum et al., 2017). In an exceptionally large study with over 1,600 measured participants the researchers also found decreased DMN FC in depressed subjects, but only in recurrent depression, not in first-episode depressed patients (Yan et al., 2019). Mixed results, revealing decreased as well as increased FC within the DMN, were also found, hinting toward abnormal DMN functioning in depression (Zhu et al., 2012; Guo et al., 2014).

In treating depression, Cognitive Behavioral Therapy (CBT) and interventions including CBT elements show the strongest evidence in the psychological field (Treatment Target: Depression, 2022). In CBT patients are taught to identify irrational beliefs and dysfunctional thought schemes that entail negative emotions or dysfunctional behavior (Beck et al., 1979) and they are supported to learn the skill of checking the validity of their (negative) beliefs and distance themselves from these beliefs (DeRubeis et al., 2008). The neural mechanisms that underlie a CBT treatment have been researched far less than the efficacy of CBT and until today have not been understood satisfyingly. In a review, DeRubeis et al. (2008) compared the neural changes in patients who received medication or Cognitive Therapy (CT^10^) and concluded: Amygdala hyperactivity in depressed patients decreased directly due to medication while in patients who received CT prefrontal hypoactivity increased due to therapy and since the prefrontal cortex inhibits the amygdala, amygdala activity decreased indirectly. In a more recent review, the authors conclude that there are indications for biological changes in the brain caused by CBT, but they are not as homogenously clear as one might wish (Franklin et al., 2016). Most commonly, a change in prefrontal areas is observed after CBT (Lueken and Hahn, 2016), specifically a deactivation in the dorsal ACC during resting state (Franklin et al., 2016). Less conclusive are the findings about changes in the PCC, parts of the prefrontal cortex and the amygdala (Franklin et al., 2016). They are still too heterogeneous to assume a model for the effects of CBT (Franklin et al., 2016). Despite the overwhelming amount of research that shows the healing effects of CBT on depression and its first attempts for neurobiological explanation, there are still patients who do not respond to CBT. Leichsenring and Steinert (2017) challenge the superiority of CBT compared to other psychotherapies and conclude that CBT should not be considered gold standard due to limited study quality, weak empirical tests and limited efficacy (response rate of about 50%; Leichsenring and Steinert, 2017). The reasons why a considerable amount of patients does not respond to CBT and their possible correlates on a neurophysiological level are yet to be found. To meet the needs of as many patients as possible and to make individualized treatment possible, it is necessary to further investigate CBT and possible alternatives, such as HT.

Alladin and colleagues developed an approach adding hypnotherapeutic elements to depression specific CBT (Alladin, 2006; Alladin and Alibhai, 2007) and found that this hypnotherapeutic addition to CBT was more efficacious than CBT alone (Alladin and Alibhai, 2007). Fuhr et al. (2021) were the first to compare HT only to CBT in depressed patients. In their study, HT included formal hypnotic inductions and self-hypnosis, as well as elements of Ericksonian HT reaching beyond formal hypnosis, namely the work with stories and metaphors, the construction of inner mental images and future visions, the activation of inner resources and biographical work (Wilhelm-Goessling et al., 2020). The authors show that depressed patients benefited equally from both therapies. HT was not inferior to CBT in terms of the extent of symptom reduction (Fuhr et al., 2021). Still, the neurophysiological correlates and mechanisms of these effects have yet to be investigated.

To conclude on the objective of this work, we built on the fact, that hypnosis/hypnotic trance has been implemented in treatments for thousands of years. On a neurophysiological level, hypnosis/hypnotic trance has been associated with the DMN, but applied in a therapeutical setting, HT has rarely been investigated. The effect of HT on the DMN has not been subject to investigation, as far as we know. Depression on the other hand, a highly prevalent disorder, has been researched repeatedly. Neurophysiologically, acutely depressed subjects show aberrant functioning of the DMN and this has been linked to cognitive processes during depression, like rumination. CBT has proven itself as an effective psychological treatment of depression and studies on its neural effects suggest alterations through therapy in regions that are also part of the DMN. In contrast, HT has never been investigated in its neural effects on depressed patients. In this study, we aimed to shed some light on the mostly unknown field of neural effects of CBT and HT for depression, specifically regarding the DMN. To this end, we conducted a neuroimaging study on 75 depressed patients undergoing either CBT or HT. Our imaging device of choice was functional near-infrared spectroscopy (fNIRS). This is a non-invasive method for optically-based functional imaging, offering many advantages. It is easy and quick to apply in a noise-free setting, is tolerant toward movement (Fallgatter et al., 2004; Ernst et al., 2012) and has no specific exclusion criteria. These advantages are of special importance when working with a clinical sample, because the study participants were more prone to stressful stimuli due to their mental condition. On the other hand, fNIRS bears disadvantages, like the fact, that near-infrared light does not penetrate the brain tissue further than 1–2 cm (Patil et al., 2011). Therefore, we focused on measuring the cortical parts of the DMN, as they have been in measured in other studies (Bulgarelli et al., 2020; Duan et al., 2020). Preliminary analyses on a sub-sample of the here presented participants revealed a cortical sub-system of the DMN, including temporal and parietal-occipital regions (Rosenbaum et al., 2017). In our study, we assumed a reduction of self-reported symptoms, independent from the therapy group mirroring a therapy effect. We hypothesized this therapeutic effect to be reflected by a change in DMN associated FC over time. We did not specify a direction for the change of FC within the DMN, due to the lack of previous research on the neural effects of HT or CBT on the DMN. Further, we were interested in the therapy-specific effects on the DMN. Again, due to the lack of previous research, the therapy-specific analyses were rather exploratory and we did not specify a direction of change. Lastly, we are interested in a direct connection between possible therapy effects on symptom level and on a cerebral level by correlating the changes in symptom reduction and FC change.

## 2 Materials and methods

### 2.1 Subjects

**Figure 1:**
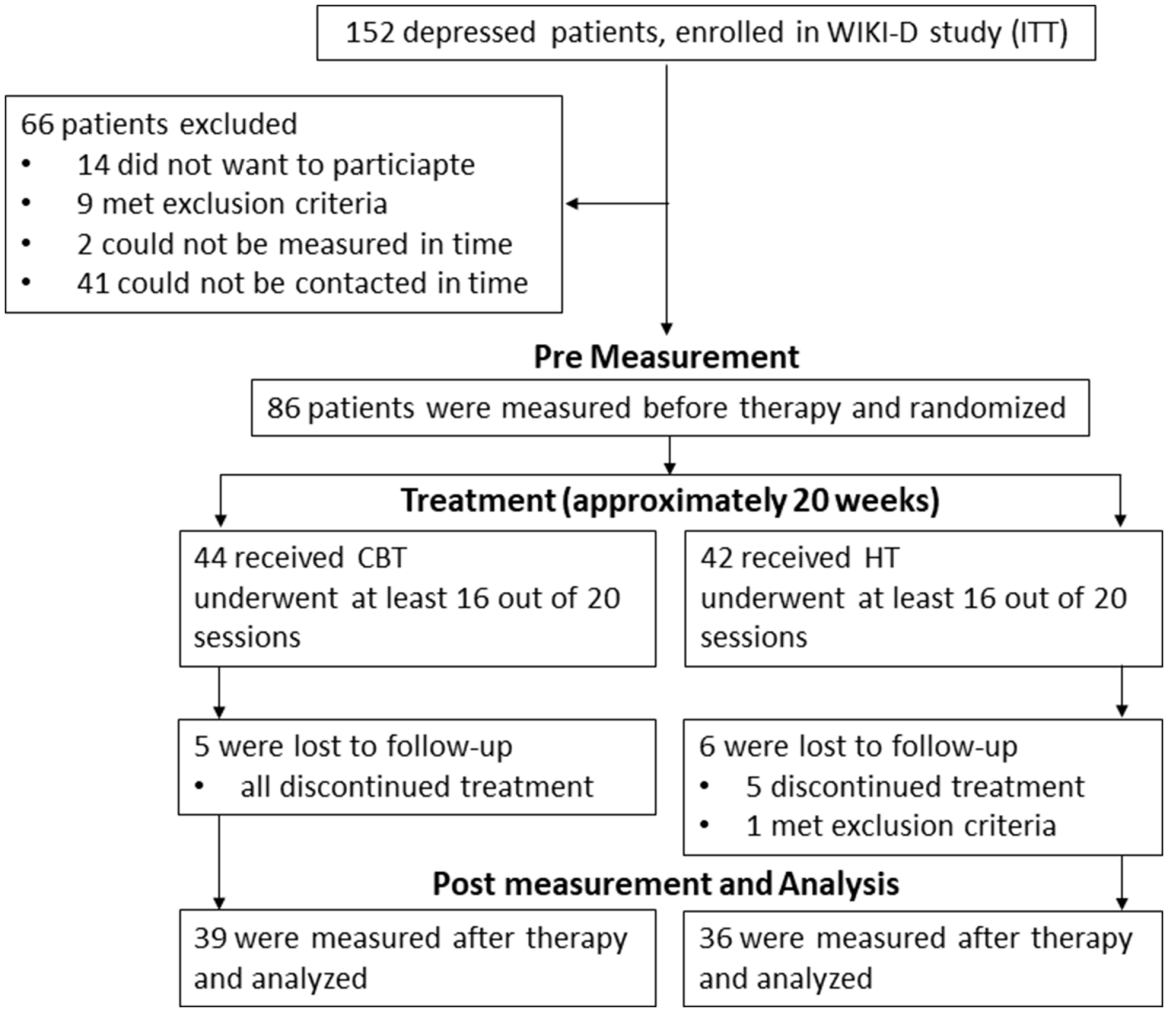
Procedure of including and measuring patients. WIKI-D study is by Fuhr et al. (2021), ITT intention to treat, CBT Cognitive Behavioral Therapy, HT Hypnotherapy.

We recruited our subject sample from the 152 depressed patients that were intended to treat (ITT^11^) in the WIKI-D study by Fuhr et al. (2021). All study participants suffered from an acute unipolar mild to moderate depressive episode. Inclusion criteria to the WIKI-D study required no change in antidepressant medication for the last 3 months and no psychotherapy in the last 12 months before the study. Trained staff used the German version of the Structured Clinical Interview for DSM-IV (SKID-I^12^; Wittchen et al., 1997) to diagnose patients. All participants of the WIKI-D study were contacted and asked to participate in our neurophysiological sub-study (Figure 1). Exclusion criteria for the neurophysiological measurements included pregnancy or nursing a child, severe neurological diseases (e.g., meningitis, epilepsy), untreated hypertension, diabetes, or other coronary diseases as well as social phobia and acute substance abuse. As a result, 75 patients (56 females, 19 males) between the age of 18 and 69 (*M* = 39.24, *SD* = 14.85) participated in both measurements, before and after therapy (Figure 1). 25 patients took at least one antidepressant substance (36% SSRIs or other including atypical antipsychotics, NaSSA, tricyclic antidepressants, hypericum, SSNRI, agomelatine, bupropion, anticonvulsive medication); 24 patients showed at least one acute comorbid disorder, 6 patients showed more than one acute comorbidity. Therapy group assignment was randomized (in CBT 39 subjects and in HT 36 subjects). The Ethics Committee at the Medical Faculty of the University of Tuebingen and University Hospital Tuebingen approved of this study (061/2015B02). All participants gave their written informed consent after reading the complete description of the study.

### 2.2 Statement of ethics

This study was approved with a positive ethics vote by the Ethics Committee of the University Hospital of Tuebingen (061/2015B02). All patients gave their written informed consent to participate in the study after they had read a study description.

### 2.3 Measures and therapies

All patients completed the 9 item Patient Health Questionnaire Depression Scale (PHQ-9^13^; Kroenke and Spitzer, 2002) to evaluate self-reported depressive symptom severity before and after therapy. The difference between pre therapy and post therapy scores were used as indicators for therapeutic change (decrease in symptoms indicate positive therapeutic effect). CBT, as well as HT, consisted of 20 sessions, and patients were treated individually. The therapy was considered completed when patients visited at least 16 of the 20 sessions, which was accomplished by 76 patients. One patient became pregnant during therapy and could therefore not be measured a second time.

CBT as well as HT were conducted by four experienced clinicians who were specifically trained for the manuals used in this study. Both manuals included The CBT manual was based on well-established manuals (Hautzinger, 2013) and included elements like psychoeducation, cognitive restructuring, behavioral activation, and the development of problem solving and interpersonal skills. The HT manual was exclusively developed for the therapy study (Fuhr et al., 2021) we based our measurements on. It aimed at activating emotions, and reinforcing personal resources. Positive visions were developed and relevant positive as well as negative biographical events were worked on. This was done with formal trance induction, as well as utilization techniques and story/metaphor telling (Wilhelm-Goessling et al., 2020).

### 2.4 Procedure

The NIRS measurement took place between the time of diagnostic procedure of the WIKI-D study (Fuhr et al., 2021) and the beginning of the psychotherapy, the latest within 1 week after the first therapy session. After the end of therapy, the second measurement was conducted, the earliest 1 week before the last session, the latest 4 weeks after the end of therapy.

The NIRS measurement itself lasted around 2 h, both at baseline and post treatment. After being seated 75 cm in front of a computer screen, the NIRS cap was placed on the subjects’ heads according to their measurements. Resting state was measured first. All subjects were instructed to close their eyes, sit still and not fall asleep during the resting state measurement. The measurement lasted 7 mins. Afterwards the patients were asked to report their experiences during resting state in an open self-report form and rate the time they spend on specific processes (e.g., relaxation) on visual analog scales (Rosenbaum et al., 2017). After that, a second (gait) and third (Emotional Stroop) paradigm were presented, the results of which are to be reported elsewhere. During the measurement oxygenated (O2Hb^14^) and deoxygenated hemoglobin (HHb^15^) were recorded continuously after a 10 s baseline measurement. All subjects received a small monetary compensation for their participation. Besides the resting state two additional paradigms were presented, the results of which are reported elsewhere (Haipt et al., 2022).

### 2.5 Near-infrared spectroscopy and regions of interest

**Figure 2:**
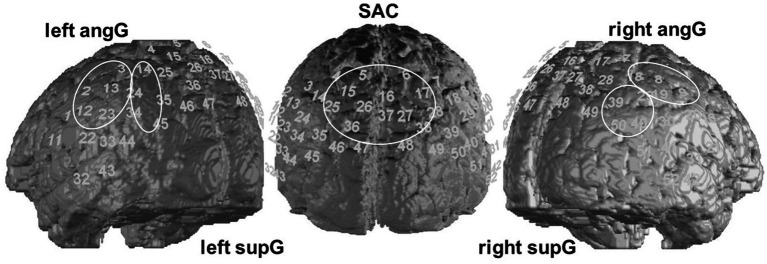
Regions of interest (ROI). We analyzed 5 ROIs, consisting of the portrayed channels, concerning the FC within and between them.

NIRS is based on the ability of light in the near-infrared spectrum to penetrate the skull and tissue. The light’s absorption depends on the oxygenation of hemoglobin in the investigated brain tissue and, therefore, the absorption rate indicates the relative concentration of O_2_Hb and HHb. This leads to conclusions on activation changes in cortical areas of the brain. In our study we used an ETG-4000 Optical Topography System (Hitachi Medical Corporation, Tokyo, Japan) using a 52-channel array of 33 optodes (17 light emitters and 16 detectors). Due to this limited amount of measuring optodes, we covered the regions of the DMN closest to the skull and being identified as sub-system of the DMN by Rosenbaum et al. (2017). These regions of interests (ROIs^16^) included parietal and temporal brain areas with a temporal resolution of 10 Hz. The localized hub nodes of this sub-network were part of the middle somatosensory association cortex (SAC^17^), left supramarginal gyrus (supG^18^) and the right angular gyrus (angG^19^). We were interested in investigating these regions including the hub nodes as well as their hemispheric counterpart. Anatomic regions were assigned to channel positions using a neuronavigation system on a volunteer’s head. This resulted in five ROIs: the SAC spreading over both hemispheres (channels 4, 5, 6, 7, 15, 16, 17, 25, 26, 27, 28, 35, 36, 37), the left angG (channels 2, 3, 12, 13, 23), right angG (channels 8, 9, 18, 19, 30), left supG (channels 14, 24, 34, 45) and right supG (channels 29, 39, 40, 50) as shown in Figure 2. The inter-optode distance was 3 cm and near-infrared light of two wavelengths (695 and 830 nm) was used. The optodes were arranged in a 3×11-rectangular shape on the subjects’ heads according to the international 10/20 System. Channel 16 was placed over Pz, the anterior channels 43 (left) and 52 (right) were positioned on the temporal electrode positions T3 and T4, respectively.

### 2.6 Data analyses

#### 2.6.1 Preprocessing

Firstly, the recorded data was preprocessed using MATLAB R2017b (MathWorks Inc., Natick, United States); brain plots were also generated with this software. This pre-processing included applying a temporal derivative distribution repair (Fishburn et al., 2019), band-pass filtering (0.1–0.01 Hz) to minimize low- and high-frequency noise, as well as the algorithm of Cui et al. (2010) for movement artifact reduction. Then all signals were visually inspected for local artifacts: Across all 75 pre measurements including 52-channels per measurement a total of 25 channels distributed over 15 subjects for the pre and 26 distributed over 13 subjects for the post measurement were interpolated, respectively. In these cases, channels were interpolated from adjacent channels. To reduce the influence of global signal changes, a global signal reduction using a spatial Gaussian kernel filter with a standard deviation of σ = 40 was applied. After preprocessing, FC-coefficients were computed with Pearson correlation after checking the variance of the channels for robustness and eliminating extreme values, then a Fishers r-to-z-transformation was conducted (Silver and Dunlap, 1987), each channel serving as seed. FC for the ROIs was computed by calculating the means of the FC coefficients belonging to this ROI.

#### 2.6.2 Statistics

Further analyses were conducted using R Studio (R Studio Inc., Boston, USA). Non-brain graphs were also produced using this program. We used linear models to test our hypotheses regarding symptom reduction assessed with the PHQ-9 as well as changes in FC from pre to post and possible interaction with groups. Marginal sums of squares were used. To further explore main or interaction effects t-tests were used to conduct post-hoc testing; means (M) and standard deviations (SD) are reported. Due to its exploratory nature regarding the therapy-specific effects we report the effects without correction for multiple testing. Still, the chance of a type I error remains due to a relatively high number of tested models (*n* = 15), therefore we do also report the *p*-values and significance levels after a correction with a modified Bonferroni method, the Armitage-Parmer method. This method is more or less conservative in correcting depending on the correlation between the data, as described in Sankoh et al. (1997). According to our main hypotheses, PHQ-9 scores (pre and post treatment) and FC scores served as outcome variables. To explore network activity, we investigated the FC between all ROIs as well as within each ROI which sums up to 15 outcome variables. As predictors served the effect coded “time” (pretreatment = 1, posttreatment = −1) and therapy approach (“group”; CBT = 1, HT = −1). In a last step, we correlated changes in symptoms and FC by using correlation tests on the change scores (post score – pre score) of the PHQ-9 and the relevant FC. For all analyses a level of significance α = 0.05 was assumed.

## 3 Results

On a behavioral level we found a therapy effect over time for all patients, displaying a significant reduction in self-reported symptoms [*β* = 3.91, *p* < 0.001, *M*(PHQ-9 pre therapy) = 14.78, *SD*(PHQ-9 pre therapy) = 3.99, *M*(PHQ-9 post therapy) = 6.70, *SD*(PHQ-9 post therapy)] = 4.37. An interaction effect of time and group did not yield significance, implying that this symptom reduction over time did not differ between the therapy groups (*β* = −0.19, *p* = 0.59). Post-hoc within-group t-testing showed significantly reduced symptoms in CBT [*t*(38) = 11.20, *p* < 0.001, *M*(PHQ-9 pre therapy) = 14.69, *SD*(PHQ-9 pre therapy) = 4.05, *M*(PHQ-9 post therapy) = 6.70, *SD*(PHQ-9 post therapy) = 3.99] and HT [*t*(35) = 8.63, *p* < 0.001, *M*(PHQ-9 pre therapy) = 14.89, *SD*(PHQ-9 pre therapy) = 3.99, *M*(PHQ-9 post therapy) = 7.25, *SD*(PHQ-9 post therapy) = 4.80].

On a neurophysiological level and exploratory in nature, we found two effects when testing time and group on 15 intra- and inter-node connections within the DMN without correcting for multiple testing. Effect.

**Figure 3:**
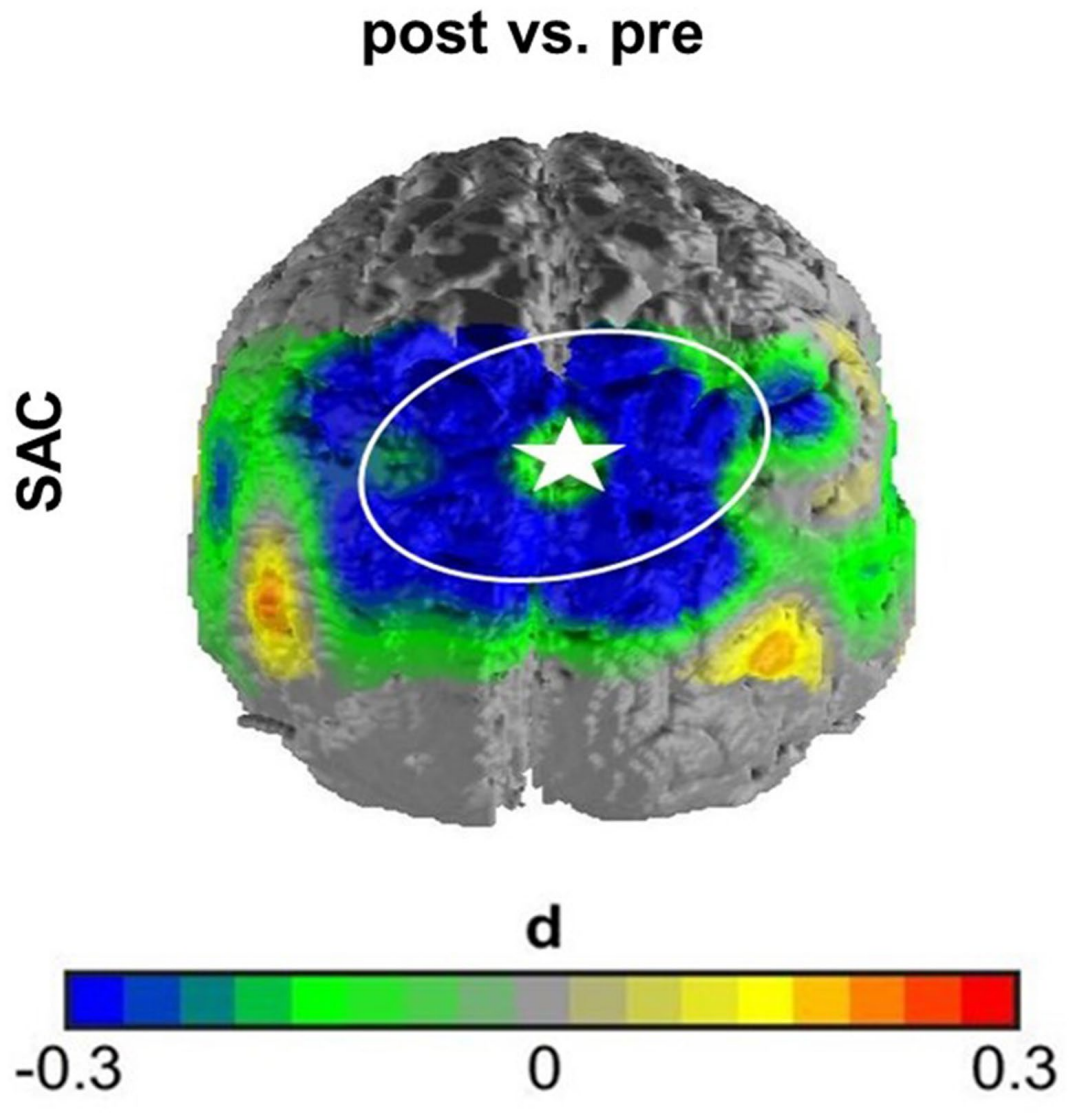
Effect size of main effect of time without correction. The FC within the SAC decreased over time comparing before therapy to after therapy. Channel 16 (marked with star) served as seed channel for this graph, displaying the contrast of correlations over time of all SAC channels to channel 16. This effect did not withstand correction for multiple testing.

**Figure 4:**
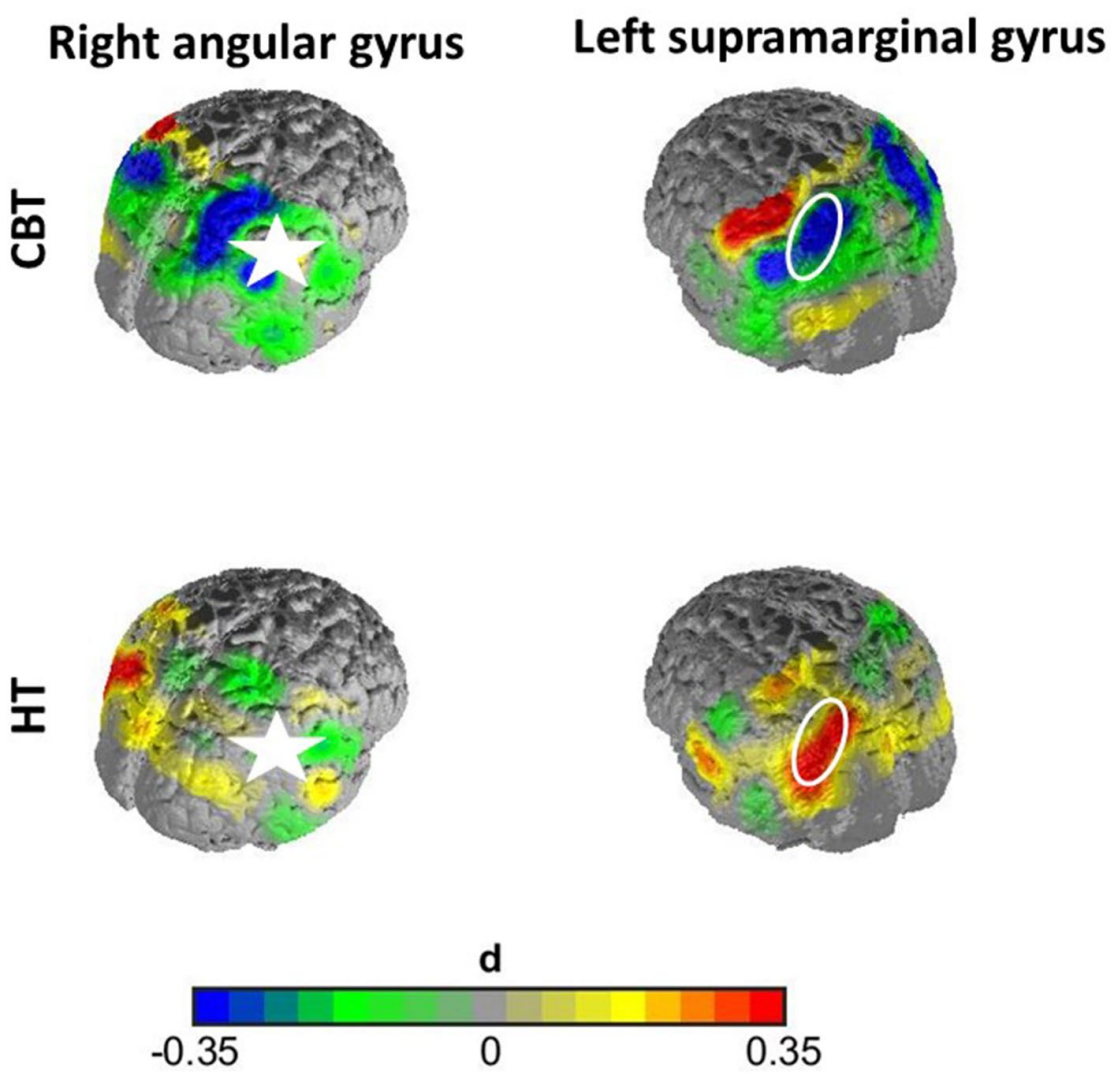
Effect size of interaction effect of time and group without correction. The FC between the right angG and left supG decreased non-significantly throughout therapy in the CBT group, while it increased in the HT group significantly. The right angG channels (marked with star) served as seed region for this graph, displaying the time-contrast of correlations of supG channels to the seed region for both groups. This effect did not withstand correction for multiple testing.

In the first case, namely the FC within the SAC, the time predictor yielded significance on an uncorrected significance level of α = 0.05, implying a change in FC within the SAC throughout therapy. FC decreased over therapy [*β* = 0.04; *p* = 0.01; *M*(post therapy) = 0.48, *SD*(post therapy) = 0.17] compared to before therapy [*M*(pre therapy) = 0.56*, SD*(pre therapy) = 0.22], as displayed in Figure 3. After Armitage-Parmar correction, this effect did not yield significance (corrected significance level α = 0.01) with *p* = 0.05. The mean Pearson correlation of the FC in the SAC and all other nodes was *r* = 0.34. A very small interaction effect of time and group yielded (uncorrected) significance (*β =* 0.03, *p* = 0.05) implying a change in FC between the right angG and left supG, differing among the therapy groups. In CBT the FC between the right angG and left supG decreased throughout therapy [*M*(pre therapy) = 0.32, *SD*(pre therapy) = 0.28], [*M*(post therapy) = 0.26, *SD*(post therapy) = 0.14], while it increased in HT [*M*(pre therapy) = 0.26, *SD*(pre therapy) = 0.19], [*M*(post therapy) = 0.32, *SD*(post therapy) = 0.15], as displayed in Figure 4. We conducted an exploratory post-hoc within-group t-tests which showed change to be only significant in the HT group (*t*(35) = −2.15, *p* = 0.04). After Armitage-Parmar correction, this effect did also not yield significance (corrected significance level α = 0.01) with *p* = 0.20, including a mean Pearson correlation of the FC of the right angG and left supG to all other nodes *r* = 0.48.

All means and SDs of the analyzed ROIs overall and separately for both groups are displayed in Supplementary Table 1.

Correlation tests between the change scores of symptoms and changes scores of the FC of the significant ROIs showed no significant correlation between the decrease of FC within the SAC throughout therapy and the symptom reduction overall (*r*(73) = − 0.87, *p* = 0.39) nor between the increase of FC between the right angG and left supG throughout therapy and the symptom reduction in the HT group (*r*(34) = 0.36, *p* = 0.72).

To sum up the results: while treatment effects could be observed on the level of depressive symptoms, they were only small trends of change to be found in two components associated with the DMN: the FC within the SAC changed throughout therapy, independent from the therapy patients received, and the FC between the right angG and left supG increased for HT patients. Both effects were only found when analyzing the data exploratorily; when the effects were corrected for multiple testing they did not yield significance anymore. Further, the symptom reduction did not correlate significantly with either of these neurophysiological changes.

## 4 Discussion

### 4.1 General discussion

In this study, we wanted to bring together the very different research objectives of DMN functioning in depression, its change throughout therapy, as well as hypnosis related DMN changes. Therefore, we investigated the neural effects of CBT and HT for depressed patients regarding key parts of the DMN. Results show that both therapies were effective in patients’ symptom reduction. When taking an exploratory look at the data, a tentative change in FC throughout therapy was observed in two components associated with the DMN. As a small effect, FC within the SAC trended to decrease with time, indifferently of the therapy group, whereas an inter-hemispheric connection, namely between the right angG and left supG, hinted toward in increase only in the HT group. Both effects did not yield significance anymore after correcting for multiple testing. A correlation between these FC changes and the symptom reduction could not be found. Considering the fact, that this study is the first of its kind (to our knowledge), we will offer first thoughts about the interpretation of possible effects.

In this study, the ROI representing the SAC included a considerable amount of channels and spread over both hemispheres, accounting for the rather small spatial resolution of fNIRS (McCormick et al., 1992; Wabnitz et al., 2010). We assume that the parietal midline channels of this ROI include cortical parts of the PCu, which from the beginning of DMN research has been shown to be an important part of this network (Raichle et al., 2001). Most recent research shows that especially midline cortical areas seem to play an important role in depression-specific DMN abnormalities (Scalabrini et al., 2020). More specifically, the authors show that the depression-specific increase of DMN FC in these midline regions can be attributed to the abnormally high connection of the DMN to other brain networks and not to an increased intra-network connection: The abnormally active DMN midline regions were strongly connected to brain regions outside the DMN. The authors conclude that this could account for the many cognitive, sensory and affective functions, which are not associated with the DMN and still impaired in depression (Scalabrini et al., 2020). Our results tentatively show a decrease of FC within the SAC over time. This might hint toward a normalization of this DMN midline component in depressed patients throughout therapy and would be in line with previous research (Anand et al., 2005; Greicius et al., 2007; Grimm et al., 2009; Sheline et al., 2009). However, we did not investigate the connection of the SAC to other core networks and further research is needed here. Further, the effect did not withstand correction for multiple testing. In future research the FC within the SAC should be investigated more thoroughly and based on specific hypotheses. Might, if the effects remain visible in further research, it might reflect an overall treatment effect not specified by certain therapy programs.

Another trend hinting toward a therapy-specific effect could be observed in an interhemispheric DMN connection. The interaction effect in the linear model did not withstand correction of multiple testing, while the post-hoc test still hints toward an increase of FC over time in the HT group. As suggested above, more research and hypotheses-based analyses are needed to validate or reject this trending effect. If it were found to be robust, the question would arise, why this increase in FC in this interhemispheric DMN connection only occurs in the HT group. Possibly, it is due to the connection between the DMN and hypnosis itself. As two authors (Halsband and Wolf, 2021) have summarized, hypnosis has been mostly associated with a decrease of DMN FC. However, the studies conducted on the DMN and hypnosis differ greatly from the study presented here. We used a clinical sample, did not control for suggestibility and the patients applied hypnosis repeatedly over approximately 4 months of HT. The previous studies in hypnosis and the DMN were conducted with healthy participants, mostly high suggestible. Furthermore, we did not induce hypnosis during or right before our DMN measurement, while this was the case in previous studies on hypnosis and the DMN (Halsband and Wolf, 2021). Thus, while in general hypnosis is associated with a decrease of FC in the DMN, a long-term effect of hypnosis applied in a clinical context could result in an increased DMN FC outside of a hypnotic state. This connection between hypnosis in a clinical context applied repeatedly and changes of the DMN have never been investigated before and much more research is needed to understand it more thoroughly.

As a group of authors (Scalabrini et al., 2020) stress the role of the DMN midline regions in depression-specific over-activation, the role of rather lateral DMN regions and their connections remains unclear. At the same time, hypnosis was shown to be connected to parts of the DMN. However, which part of the DMN plays which role during hypnosis is still unknown. Consistently, involvement of prefrontal parts and the PCC during hypnotic trance has been reported (Rainville et al., 2002; Egner et al., 2005; Cojan et al., 2009; McGeown et al., 2009; Pyka et al., 2011; Deeley et al., 2012); lateral parts of the DMN were associated with hypnotic trance in one case (Pyka et al., 2011). Functionally, the DMN has been linked to a self-referential, introspective state, which may have very plausibly been fostered through the HT interventions. In HT, a major technique involved inducing pleasant emotions linked to personal experiences to make the patients feel strong, competent, and hopeful (Wilhelm-Goessling et al., 2020). If there a HT-specific effect on the DMN was found to be robust, it could be a first indication of a HT-specific impact on this network, which has been linked to self-referential processing. Further, the results of a second paradigm used in this study showed changed activity in the superior temporal sulcus in the HT group. This effect was moderated by rumination (Haipt et al., 2022). From these results we suggest an indirect involvement of the DMN, reflected by rumination on a symptom level, and temporal activity which is associated with emotional processing, that only occurred in the HT group (Haipt et al., 2022). Lastly.

Further, it is crucial to point out, that hypnosis cannot only be understood as a distinct and separate state of consciousness, as defined by some authors (Spiegel and Moore, 1997). Rather, there are many factors that relate to hypnosis and a person’s response to it (Jensen et al., 2015; Geagea et al., 2024) as well as different mechanisms that a person’s response to hypnosis is based on Zahedi et al. (2024). It is very likely that these different mechanisms and factors moderate or mediate the neurophysiological effect of hypnosis and this might also explain (some of) the heterogeneity of results concerning the effect of hypnosis on the DMN (regarding the direction of FC change within the DMN as well as which part of the DMN is involved). This could also possibly explain, why we only found a trend for an effect in the HT group. As our results show on symptom level, the patients in the HT group did respond to this therapy and their symptoms reduced. However, the way they responded to the therapy, the formal trances as well as the informal suggestions, metaphors etc., might have differed greatly. Biological, cognitive, and social factors (Jensen et al., 2015) as well as the underlying mechanisms (Zahedi et al., 2024) might have affected the patient’s response to the therapy and thus the amount and the direction of change of related DMN activity. Klicken oder tippen Sie hier, um Text einzugeben. Therefore, in future analyses the effects of hypnosis on the DMN should be looked at more individually, accounting for the different factors and mechanisms influencing a person’s response to hypnosis. With our study, we could only open a door to research that aims at understanding the specific effects of HT on the DMN. Much more research is needed, to gain first answers.

Why the therapeutic effect of CBT, which was clearly visible on a symptom level, was not reflected in DMN-related FC should be investigated in the future. Possibly, CBT specific effects can be found more easily in cerebral networks that are associated with high-level cognitive functioning like the Central Executive Network (CEN^21^; Menon, 2011). This network could not be investigated in the setup we chose for this study.

The lack of correlations between symptom reduction and DMN associated changes might hint toward a multi-faceted connection between DMN activity on a cerebral level and symptoms on a behavioral level. As the authors of one study suggest (Scalabrini et al., 2020), many symptoms in depression are associated with processes of non-DMN networks like movement, memory, reward, perception, and they might be connected to the DMN on a neurophysiological level through over-connections with the DMN. IN our study we did not measure the connection between the DMN and other core networks. It is plausible that symptoms and abnormal DMN activity in depression are both components of a very complex psychopathology but do not relate directly or linearly. Moderating or mediating factors such as subtypes of depression, amount of rumination, extent of somatic syndrome in depression, symptom severity or medication seem likely to be part of the equation. In a review, the authors report that in different studies up to five depression subtypes, which differed on a symptom as well as brain network FC level, were identified (Chahal et al., 2020). Also, non-linear relations could draw another connection between symptoms and DMN activity. In future research, variations in symptoms should be controlled for. Also, NIRS measurements should be conducted more often (e.g., weekly) to find possible non-linear relations.

Further, it remains unclear if psychotherapy and DMN changes relate directly or if they are also moderated or mediated by other factors. Psychotherapy, in the case of this study CBT and HT, includes many different techniques, aspects, a unique relationship between therapist and client and it is temporally spread—in our case over half a year—with weekly sessions, which leaves much time to process, learn and apply aspects of therapy. So far, few studies have been conducted on the effects of therapy on the DMN; in one, using functional magnetic resonance imaging (fMRI^22^), the authors investigated the effect of behavioral activation on the change of the DMN and found a reduction of FC in an anterior subnetwork of the DMN after the intervention (Yokoyama et al., 2017). In another study from the same year, the authors also found a reduction of frontal DMN FC, namely between the mPFC and ACC after CBT and correlating positively with symptom reduction (Yoshimura et al., 2017). Until now it is unclear, which role temporal and parietal parts of the DMN play in therapy related DMN changes and calls for further investigation. Furthermore, time itself plays a crucial role in the progression of depression and its effects on the DMN are yet to be investigated. A clearer picture of the role of the DMN during a temporally spread therapy could be obtained by an increased frequency of measurements, e.g., weekly measurements.

### 4.2 Limitations and future research

A clear limitation to fNIRS is the restricted penetration depth of 3–5 mm into the adult cortex (Gervain et al., 2011) and thus the inability to measure subcortical processes. Especially concerning the DMN, subcortical regions are of interest (Raichle et al., 2001), like the PCC and ACC. Therefore, data from alternative imaging methods, such as fMRI, should be analyzed to underpin our findings. Further, our data on a symptom level consisted of self-reports and not an objective measure on a behavioral level or clinician administered scales. In future research a measure mirroring DMN processes, like rumination scales, should be included in the analysis. Additionally, the self-report questionnaire PHQ-9 consisted of only 9 questions, which is too short and imprecise to draw a more differentiated picture of depression symptoms and possible connections to the DMN. Another factor to consider in future research is the influence of medication. As the authors find in their very large study, the medication treatment of depressed patients was associated with decreased DMN FC, while illness duration did not play a significant role (Yan et al., 2019). Also, symptom severity was associated with reduced DMN FC only in recurrent depression (Yan et al., 2019). These factors were not included in our analyses. We suggest including the factor “medication” and the number of earlier episodes in future research on depression-specific DMN FC. Another limitation is that we derived our hypotheses concerning HT from research on hypnosis and studies, that compared hypnosis to no-hypnosis conditions. However, we looked at the long-term effects of hypnosis used in therapy and did not explicitly measure the patients during hypnosis. Further, in the previous hypnosis studies on the DMN, neither personal, nor emotional content was included, nor was hypnosis applied repeatedly over a longer period of time, nor with patients, but healthy controls. Instead they included, e.g., relaxation (Deeley et al., 2012) or hand paralysis (Cojan et al., 2009; Pyka et al., 2011). Thus, the hypnotic suggestions used in previous studies and used in our studies are probably very difficult to compare. So, in future studies hypnotic trances similar to the ones used in a therapeutic context containing personal, emotionally relevant content, should be investigated regarding their connection to the DMN. Also, the effect of hypnotic trances applied repeatedly over a larger period of time, should be researched.

## 5 Conclusion

In this original study we investigated 75 depressed patients receiving either CBT or HT regarding FC associated with the DMN. All patients reported significantly fewer symptoms after therapy. Exploratory findings hint toward treatment effects in two components of the DMN, one independent from the therapy group, one in the HT group. These effects did not withstand correction for multiple testing and thus can hardly be interpreted. Still, even though both therapy approaches helped the patients (i.e., reduced depressive symptoms), they might have done so based on different neural mechanisms. This study serves as first insight into possible different neural mechanisms of HT and CBT and should serve as stepstone for further research.

## Data availability statement

The raw data supporting the conclusions of this article will be made available by the authors, without undue reservation.

## Ethics statement

The studies involving humans were approved by Ethics Committee at the Medical Faculty of the University of Tuebingen and University Hospital Tuebingen (061/2015B02). The studies were conducted in accordance with the local legislation and institutional requirements. The participants provided their written informed consent to participate in this study.

## Author contributions

AH: Conceptualization, Data curation, Formal analysis, Investigation, Methodology, Project administration, Visualization, Writing – original draft, Writing – review & editing. DR: Data curation, Formal analysis, Methodology, Software, Writing – review & editing. KF: Project administration, Resources, Writing – review & editing. AB: Funding acquisition, Supervision, Writing – review & editing. A-CE: Conceptualization, Formal analysis, Funding acquisition, Methodology, Project administration, Resources, Supervision, Validation, Writing – review & editing.

## Glossary

^1^HT: Hypnotherapy
^2^DMN: Default Mode Network
^3^FC: Functional Connectivity
^4^CBT: Cognitive Behavioral Therapy
^5^fNIRS: functional near-infrared-spectroscopy
^6^PCC: posterior cingulate cortex
^7^PCu: precuneus
^8^BOLD: blood oxygen level dependent
^9^ACC: anterior cingulate cortex
^10^CT: Cognitive Therapy
^11^ITT: Intention to treat
^12^SKID-I: Structured Clinical Interview for DSM-IV
^13^PHQ-9: 9 item Patient Health Questionnaire Depression Scale
^14^O2Hb: Oxygenated hemoglobin
^15^HHb: deoxygenated hemoglobin
^16^ROIs: regions of interests
^17^SAC: somatosensory association cortex
^18^supG: supramarginal gyrus
^19^angG: angular gyrus
^20^TDDR: temporal derivative distribution repair
^21^CEN: Central Executive Network
^22^fMRI: functional magnetic resonance imaging
