# Supplementary material for: Differential effects of hypnotherapy and cognitive behavioral therapy on the default mode network of depressed patients
Source: Front Psychol. 2024 Jun 27;15:1401946. doi: 10.3389/fpsyg.2024.1401946 (PMC11238146; doi:10.3389/fpsyg.2024.1401946)
Supplement: Supplementary file 1 [file Table_1.DOCX]

***Supplementary material***

**Supplementary Table**

| **FC between** | | *Mean* Pre FC | *Mean* Post FC | *SD*  Pre FC | *SD* Post FC |  |
| --- | --- | --- | --- | --- | --- | --- |
| angG left to angG left | Overall  CBT  HT | 0.33  0.31  0.35 | 0.31  0.28  0.35 | 0.19  0.19  0.19 | 0.17  0.13  0.20 |  |
| angG left to angG right | Overall  CBT  HT | 0.30  0.29  0.30 | 0.30  0.28  0.31 | 0.17  0.18  0.17 | 0.14  0.14  0.14 |  |
| angG left tp supG left | Overall  CBT  HT | 0.34  0.35  0.34 | 0.35  0.33  0.38 | 0.19  0.17  0.21 | 0.17  0.15  0.18 |  |
| angG left to supG right | Overall  CBT  HT | 0.23  0.22  0.24 | 0.25  0.23  0.27 | 0.19  0.17  0.21 | 0.15  0.15  0.15 |  |
| angG left to SAC | Overall  CBT  HT | 0.31  0.32  0.31 | 0.29  0.27  0.30 | 0.19  0.14  0.23 | 0.15  0.15  0.15 |  |
| angG right to angG right | Overall  CBT  HT | 0.34  0.35  0.33 | 0.29  0.29  0.28 | 0.19  0.21  0.17 | 0.15  0.15  0.15 |  |
| angG right to supG left | Overall  CBT  HT | 0.29  0.32  0.26 | 0.29  0.26  0.32 | 0.24  0.28  0.19 | 0.15  0.14  0.15 |  |
| angG right to supG right | Overall  CBT  HT | 0.36  0.37  0.34 | 0.32  0.30  0.35 | 0.22  0.26  0.17 | 0.15  0.16  0.14 |  |
| angG right to SAC | Overall  CBT  HT | 0.32  0.33  0.30 | 0.28  0.25  0.32 | 0.21  0.22  0.20 | 0.14  0.14  0.13 |  |
| supG left to supG left | Overall  CBT  HT | 0.43  0.45  0.41 | 0.44  0.43  0.44 | 0.21  0.23  0.19 | 0.16  0.16  0.17 |  |
| supG left to supG right | Overall  CBT  HT | 0.48  0.50  0.46 | 0.46  0.47  0.46 | 0.21  0.25  0.17 | 0.17  0.16  0.18 |  |
| supG left to SAC | Overall  CBT  HT | 0.46  0.46  0.46 | 0.44  0.43  0.45 | 0.20  0.22  0.18 | 0.15  0.15  0.15 |  |
| supG right to supG right | Overall  CBT  HT | 0.51  0.52  0.51 | 0.46  0.46  0.46 | 0.19  0.20  0.18 | 0.16  0.16  0.17 |  |
| supG right to SAC | Overall  CBT  HT | 0.43  0.42  0.44 | 0.41  0.39  0.44 | 0.20  0.23  0.17 | 0.14  0.14  0.13 |  |
| SAC_SAC | Overall  CBT  HT | 0.56  0.53  0.59 | 0.48  0.46  0.50 | 0.21  0.22  0.19 | 0.17  0.18  0.15 |  |

**Supplementary Table 1.** All means and standard deviations for all 15 ROIs, overall and both groups separately. *FC* functional connectivity, *HT* Hypnotherapy, *CBT* Cognitive Behavioral Therapy, *angG* angular gyrus, *SAC* somatosensory association cortex, *supG* supramarginal gyrus.
